# Malignant Ascites Promote Adhesion of Ovarian Cancer Cells to Peritoneal Mesothelium and Fibroblasts

**DOI:** 10.3390/ijms22084222

**Published:** 2021-04-19

**Authors:** Paweł Uruski, Justyna Mikuła-Pietrasik, Martyna Pakuła, Sylwia Budkiewicz, Marcin Drzewiecki, Andrey N. Gaiday, Małgorzata Wierzowiecka, Eryk Naumowicz, Rafał Moszyński, Andrzej Tykarski, Krzysztof Książek

**Affiliations:** 1 Department of Hypertensiology, Poznan University of Medical Sciences, Długa 1/2 Str., 61-848 Poznan, Poland; puruski@ump.edu.pl (P.U.); mpakula@ump.edu.pl (M.P.); malgorzata.wierzowiecka@gmail.com (M.W.); tykarski@o2.pl (A.T.); 2 Department of Pathophysiology of Ageing and Civilization Diseases, Poznan University of Medical Sciences, Długa 1/2 Str., 61-848 Poznan, Poland; jmikula@ump.edu.pl (J.M.-P.); sbudkiewicz@ump.edu.pl (S.B.); mdrzewiecki@ump.edu.pl (M.D.); 3 Department of Obstetrics and Gynecology, West Kazakhstan Marat Ospanov Medical University, 50B 12th Microdistrict Apt.21, Aktobe 030008, Kazakhstan; a.gaiday@mail.ru; 4 General Surgery Ward, Medical Centre HCP, 28 Czerwca 1956 r. 223/229 Str., 61-485 Poznan, Poland; eryknaumowicz777@gmail.com; 5 Division of Gynecological Surgery, Poznan University of Medical Sciences, Polna 33 Str., 60-535 Poznan, Poland; rafalmoszynski@gmail.com

**Keywords:** cancer cell adhesion, malignant ascites, ovarian cancer, peritoneal metastases

## Abstract

Although malignant ascites (MAs) are known to contribute to various aspects of ovarian cancer progression, knowledge regarding their role in the adhesion of cancer cells to normal peritoneal cells is incomplete. Here, we compared the effect of MAs and benign ascites (BAs) on the adhesion of A2780 and OVCAR-3 cancer cells to omentum-derived peritoneal mesothelial cells (PMCs) and peritoneal fibroblasts (PFBs). The results showed that MAs stimulated the adhesion of A2780 and OVCAR-3 cells to PMCs and PFBs more efficiently than did BAs, and the strongest binding occurred when both cancer and normal cells were exposed to the fluid. Intervention studies showed that MAs-driven adhesion of A2780 cells to PMCs/PFBs depends on the presence of TGF-β1 and HGF, whereas binding of OVCAR-3 cells was mediated by TGF-β1, GRO-1, and IGF-1. Moreover, MAs upregulated α5β1 integrin expression on PFBs but not on PMCs or cancer cells, vimentin expression in all cells tested, and ICAM-1 only in cancer cells. When integrin-linked kinase was neutralized in PMCs or PFBs, cancer cell adhesion to PMCs and PFBs decreased. Collectively, our report shows that MAs may contribute to the early stages of ovarian cancer metastasis by modulating the proadhesive interplay between normal and cancer cells.

## 1. Introduction

Ovarian cancer, the most lethal gynecological malignancy [1], preferentially metastasizes into the peritoneal cavity [2]. The process of intraperitoneal dissemination of ovarian cancer is a highly complex and multistage phenomenon, and several elements are driven by malignant ascites (MAs), a peritoneal, inflammatory fluid that accumulates in excess in a large group of ovarian cancer patients [3]. MAs carry the cancer cells within the peritoneum after their detachment from the ovary [4], generate an immunosuppressive milieu that protects the malignant cells [5], stimulate a tissue vascularization by secreting a variety of proangiogenic proteins [6], promote epithelial–mesenchymal transition (EMT), facilitating transmesothelial invasion of cancer cells [7], and induce premature senescence of normal peritoneal mesothelial cells (PMCs), forcing them to develop a cancer-promoting phenotype [8]. At the same time, very little is known about the effect of MAs on other critical aspects of ovarian cancer cell metastasis, such as their adhesion to PMCs and peritoneal fibroblasts (PFBs).

According to the current knowledge, both PMCs and PFBs actively stimulate cancer cell adhesion [9,10]. This process is controlled by reciprocal interactions between different surface ligands and receptors, of which α_5_β1 integrins interacting with extracellular matrix (ECM) proteins [11,12] in cooperation with integrin-linked kinase (ILK) [13] play a prominent role. Considering that the adhesion of ovarian cancer cells may also depend on some soluble agents present in the peritoneal environment (e.g., lysophosphatidic acid [14]), we asked whether MAs may also contain factors that could intensify this process, and if so, what is the mechanism of the proadhesive outcome of MAs activity in terms of soluble mediators, cell-surface molecules, and signaling pathways contributing to this phenomenon.

## 2. Results

### 2.1. Malignant Ascites Stimulate Adhesion of Ovarian Cancer Cells to PMCs and PFBs

**Figure 1:**
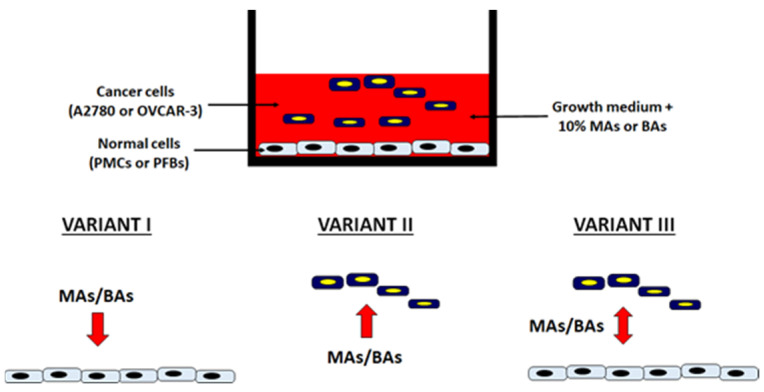
Three variants of cell exposure to malignant ascites (MAs) or benign ascites (BAs) during the analysis of ovarian cancer cell adhesion to the normal peritoneal cells. Red arrows indicate a direction of the fluids activity. PMCs: peritoneal mesothelial cells; PFBs: peritoneal fibroblasts.

The adhesion of cancer cells to PMCs or PFBs was tested under the following conditions: normal cell exposure to MAs/BAs (benign ascites), cancer cell exposure to MAa/BAa, and exposure of normal and cancer cells to MAs/BAs (Figure 1).

**Figure 2:**
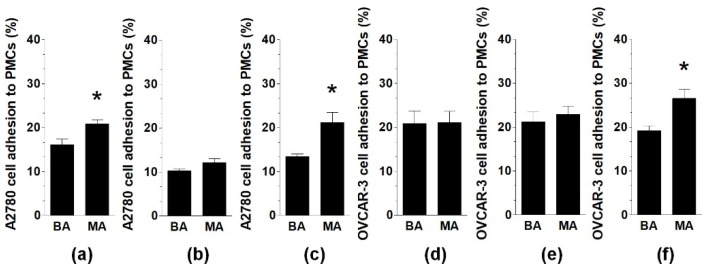
Effect of MAs and BAs on ovarian cancer cell (A2780, OVCAR-3) adhesion to PMCs. Three strategies of adhesion measurements were employed: exposure of PMCs to MAs or BAs (adherence of untreated cancer cells) (**a**,**d**); exposure of cancer cells to MAs or BAs (adherence to untreated PMCs) (**b**,**e**); and exposure of PMCs and cancer cells to MAs or BAs (**c**,**f**). Experiments were performed using pooled PMCs from six different donors and samples of BAs and MAs from eight different patients. The results are expressed as the means ± SEMs. * *p* < 0.05 vs. BAs.

Experiments using A2780 cells showed that when the fluids were applied to PMCs alone or with cancer cells, their adhesion to PMCs in the presence of MAs was stronger than that in the presence of BAs. Regarding OVCAR-3 cells, MAs stimulated their adhesion to PMCs only when both normal and cancer cells were exposed to the fluid (Figure 2).

**Figure 3:**
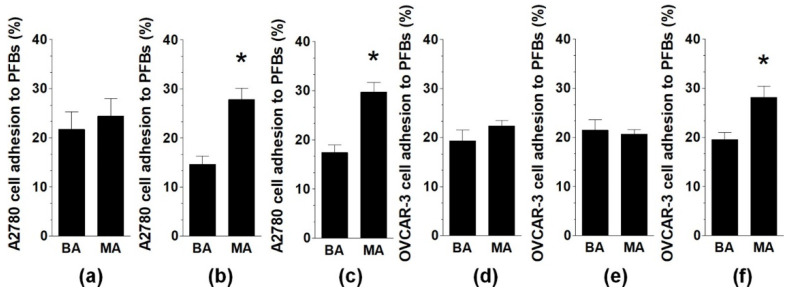
Effect of MAs and BAs on ovarian cancer cell (A2780, OVCAR-3) adhesion to PFBs. Three strategies of adhesion measurements were employed: exposure of PFBs to MAs or BAs (adherence of untreated cancer cells) (**a**,**d**); exposure of cancer cells to MAs or BAs (adherence to untreated PFBs) (**b**,**e**); and exposure of both normal and cancer cells to MAs or BAs (**c**,**f**). Experiments were performed using pooled PFBs from six different donors and samples of BAs and MAs from eight different patients. The results are expressed as the means ± SEMs. * *p* < 0.05 vs. BAs.

Analysis of the same phenomenon with PFBs revealed that A2780 adhered more efficiently to PFBs when cancer cells either alone or with normal cells were incubated with MAs. Regarding OVCAR-3 cells, MAs stimulated their adhesion to PFBs only when either PFBs or cancer cells were exposed to the fluid (Figure 3).

### 2.2. Soluble MAs-Derived Proteins Are Responsible for the Proadhesive Potential of the Fluid

**Figure 4:**
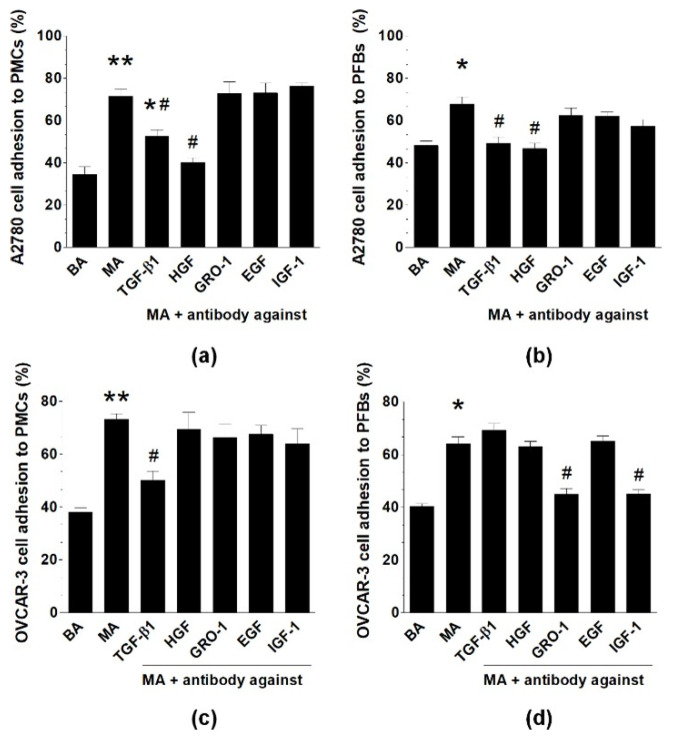
Identification of MAs-derived mediators of increased ovarian cancer cell adhesion to PMCs (**a**,**c**) and PFBs (**b**,**d**). The MAs and BAs groups indicate the variant in which both normal and cancer cells were exposed to MAs or BAs. MAs was preincubated with specific neutralizing antibodies for 4 h before it was used for the adhesion assay. Experiments were performed using pooled PMCs and PFBs from six different donors and samples of BAs and MAs from eight different patients. The results are expressed as the means ± SEMs. * *p* < 0.05; ** *p* < 0.01 vs. BAs, ^#^ *p* < 0.05 vs. MAs.

Neutralizing antibodies against five arbitrarily selected pleiotropic proteins present in MAs (TGF-β1, HGF, EGF, IGF-1, GRO-1 [8,15]) were used to check whether inhibition of these agents translates to decreased adhesion of cancer cells to PMCs or PFBs. The group in which both normal and cancer cells were exposed to MAs was used as the reference. When A2780 cells were analyzed, their adhesion to either PMCs or PFBs, which was initially increased by MAs, was reduced to values similar to those of the BAs group when TGF-β1 and HGF were neutralized. Regarding OVCAR-3 cells reacting with PMCs, MAs-stimulated adhesion was inhibited upon the neutralization of TGF-β1, whereas their interactions with PFBs were prevented in response to the neutralization of GRO-1 and IGF-1 (Figure 4).

**Figure 5:**
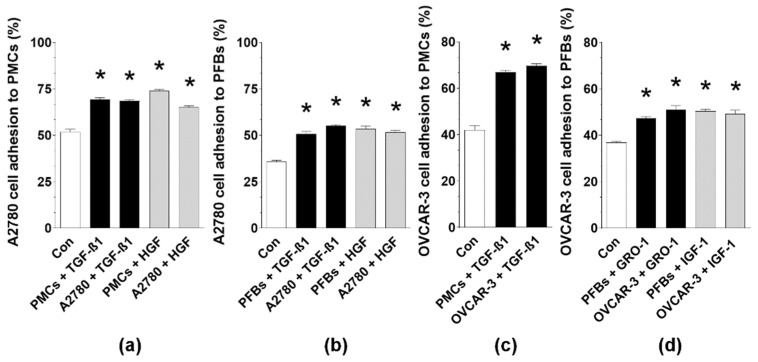
Efficiency of cancer cell adhesion to PMCs (**a**,**c**) and PFBs (**b**,**d**) in the presence of exogenous, recombinant TGF-β1, HGF, GRO-1, and IGF-1. Experiments were performed using pooled PMCs and PFBs from eight different donors. The results are expressed as the means ± SEMs. * *p* < 0.05 vs. Con.

Experiments using exogenous, recombinant forms of TGF-β1, HGF, GRO-1, and IGF-1 applied to respective cells at doses corresponding to their concentration in MAs provided clarification that both normal and cancer cells are sensitive to the identified mediators, which makes them responsible for the MAs-dependent increase in cancer cell adhesion (Figure 5).

### 2.3. Analysis of α5β1 Integrin and Integrin-Linked Kinase Engagement

**Figure 6:**
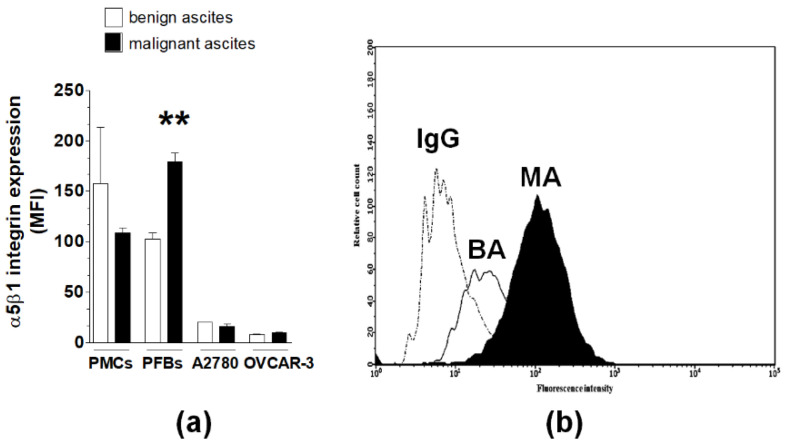
Flow cytometry analysis of α5β1 integrin expression on normal and cancer cells treated with MAs and BAs. Average expression of α5β1 integrins was quantified using GuavaSoft (**a**). Representative histogram showing the effect of MAs and BAs on α5β1 integrin levels in PFBs (**b**). Experiments were performed using pooled PMCs and PFBs from six different donors and samples of BAs and MAs from six different patients. The results are expressed as the means ± SEMs. ** *p* < 0.01 vs. BAs. MFI: mean fluorescence intensity.

Flow cytometry was used to determine changes in α5β1 integrin levels on the surface of PMCs, PFBs, A2780, and OVCAR03 cells after treatment with MAs and BAs. The analysis showed that MAs increased the expression of α5β1 integrins on PFBs but had no effect on the levels of these molecules on PMCs and cancer cells (Figure 6).

**Figure 7:**
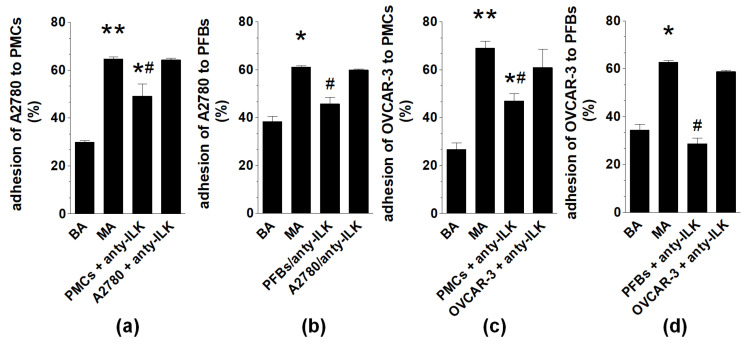
The role of ILK in MAs-dependent adhesion of ovarian cancer cells to PMCs (**a**,**c**) and PFBs (**b**,**d**). The MAs and BAs groups indicate the variant in which both normal and cancer cells were exposed to MAs or BAs. Cells were preincubated with an ILK inhibitor (Cpd22) for 3 h before exposure to MAs or BAs and subsequent adhesion assays. Experiments were performed using pooled PMCs and PFBs from six different donors and samples of BAs and MAs from eight different patients. The results are expressed as the means ± SEMs. * *p* < 0.05; ** *p* < 0.01 vs. BAs, ^#^ *p* < 0.05 vs. MAs.

When all four tested cell types were preincubated with Cpd22, an inhibitor of ILK, before MAs exposure and the subsequent adhesion assay, the efficacy of MAs-stimulated adhesion was significantly diminished when the ILK pathway was blocked in PMCs and PFBs but not in A2780 or OVCAR-3 cells (Figure 7).

### 2.4. MAs Alter the Expression of Surface Adhesion Molecules

**Figure 8:**
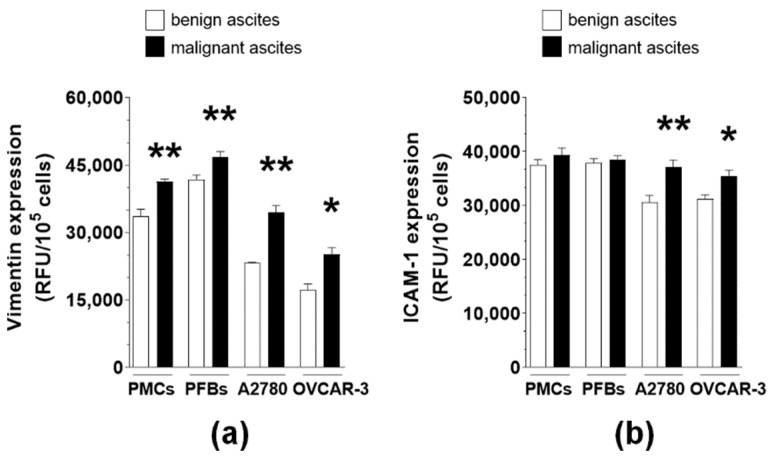
Effect of MAs and BAs on the expression of vimentin (**a**) and ICAM-1 (**b**) in normal and cancer cells. Experiments were performed using pooled PMCs and PFBs from six different donors and samples of BAs and MAs from eight different patients. The results are expressed as the means ± SEMs. * *p* < 0.05; ** *p* < 0.01 vs. BAs. RFU: relative fluorescence units.

The same experimental setup conducted for α5β1 integrin detection was used to quantify the expression of two surface molecules involved in various aspects of cell adhesion: vimentin and ICAM-1. The immunofluorescence analysis showed that MAs upregulated vimentin expression in all four cell types, whereas the induction of ICAM-1 expression was evident only in cancer cells (Figure 8).

## 3. Discussion

Because intraperitoneal carcinomatosis is responsible for fatal prognosis in ovarian cancer patients, elucidating all cellular and molecular events contributing to the aggressiveness of cancer cells in this body space is of critical importance from both predictive and therapeutic perspectives. This assumption also applies to the presence and activity of MAs, whose tumorigenic activity has already been well established [16].

In this study, we have showed that MAs stimulate the adherence of two representative ovarian cancer cell lines (A2780 and OVCAR-3) to PMCs and PFBs more efficiently than does BAs from noncancerous patients. The fundamentals for this finding have been laid out by a study in which MAs evoked premature senescence of PMCs, which translated to increased adhesion of cancer cells [8]. Here, we extended these observations by showing that MAs clearly promote the adhesion of cancer cells to young (first to second passage) normal cells and that the most profound adhesion occurs when both normal and cancer cells are subjected to MAs, similar to what plausibly happens in the peritoneal cavity in vivo.

Mechanistically, senescence-related stimulation of cancer cell adhesion to PMCs was linked with some MAs-induced changes in the secretory phenotype of normal cells, particularly the overproduction of hyaluronic acid [8]. Our intervention studies using specific neutralizing antibodies and exogenous, recombinant forms of proteins showed that improved adhesion of cancer cells is driven by certain cytokines present in MAs. The list of mediators, including TGF-β1, HGF, GRO-1, and IGF-1, depends on cancer and normal cell types. These variations, manifesting in almost all the results obtained in this study, should not be surprising given the different histological origins of A2780 (endometrioid) and OVCAR-3 cells (high-grade serous) [17], differences in their *P53* status (wild type in A2780, mutated in OVCAR-3 [18]) and estrogen receptor (ER) status (ER(-) A2780, ER(+) OVCAR-3 [19]), and different morphologies, functions, and roles of PMCs and PFBs in metastasis [20]. Notably, interactions of both cancer cell lines with PMCs and of A2780 cells with PFBs were uniformly driven by TGF-β1, which is known to promote the production of fibronectin [12], a ligand for α5β1 integrins [11,12]. Regarding the remaining proteins, HGF, GRO-1, and IGF-1 have been found to stimulate the adhesion of breast cancer [21], gastric cancer [22], and multiple myeloma cells [23], respectively; in all these studies, the activity of these proteins was dependent on β1-integrin. The validity of our findings is also supported by the fact that MAs-derived IGF-1, which is known to cooperate with estrogen-dependent signaling [24] and leads to stabilization of β1 integrins upon the stimulation of IGF-1R [25], intensified the adhesion of ER(+) OVCAR-3 cells but not ER(-) A2780 cells.

Regarding α5β1 integrins, which play a critical role in ovarian cancer cell adhesion [12] and whose inhibition acts antimetastatically [26], MAs induced their overexpression exclusively on PFBs. Taking into account that inhibition of ILK, the enzyme that transmits signals upon integrin stimulation [13], in both PMCs and PFBs reduced cell adhesion, one may theorize that the MAs-driven adhesion of cancer cells to PFBs occurs via the α5β1 integrin-ILK axis, whereas their improved binding to PMCs involves ILK and receptors other than α5β1 integrins.

The involvement of the α5β1 integrin-ILK axis was ruled out on the cancer cell side owing to the eventual changes of other adhesion-associated molecules. These include vimentin and/or ICAM-1, whose expression was elevated in MAs-treated cancer and normal cells. In fact, TGF-β1 and HGF—the pleiotropic cytokines that drive the improved adhesion of A2780 and OVCAR-3 cells in the presence of MAs—can upregulate the expression of vimentin [27,28], an intermediate filament protein whose assembly into focal adhesions is a key determinant of cell adhesion intensity [29]. Indeed, vimentin was found to control the adhesion of breast [30] and lung [31] cancer cells by modulating the activity of focal adhesion kinase. TGF-β1 also induces the production of ICAM-1 [32], whose proadhesive capacity was revealed in colorectal and pancreatic cancer cells interacting with PMCs [33].

In conclusion, our study shows that MAs contribute to the early stages of intraperitoneal ovarian cancer metastasis by promoting the adhesion of cancer cells to PMCs and PFBs. At the same time, our findings indicate that MAs-stimulated adhesion of ovarian cancer cells to normal cells is not a linear phenomenon but instead a complex network of interactions involving various triggers, surface receptors, and signaling routes. Any attempts to narrow these relationships to one probable and/or universal mechanism will fail because of the biochemical diversity of ascites [15] and the genetic differences among cancer cells [34]. Further research is also needed to identify which of the molecules identified here may constitute potential targets to therapeutically disrupt the proadhesive capabilities of MAs.

## 4. Materials and Methods

### 4.1. Materials

Unless otherwise stated, all chemicals and plastics were obtained from Sigma-Aldrich (St. Louis, MO, USA). Neutralizing antibodies against EGF (# AF236), GRO-1 (# MAB275R), HGF (# MAB294), IGF-1 (# AF-291-NA), and TGFβ1 (# AF-101-NA), as well as the antibody detecting ICAM-1 (# BBA3), were obtained from R&D Systems (Abingdon, UK). Anti-integrin α5β1 antibody (# AB1999) and isotype control antibody (# CBL600) were purchased from Merck (Dorset, UK). Antibodies against vimentin (# 5741) were obtained from Cell Signaling Technology (Beverly, MA, USA). Integrin-linked kinase inhibitor (Cpd22) was purchased from Sigma-Aldrich. Exogenous, recombinant human TGF-β1, HGF, GRO-1, and IGF-1 were obtained from R&D Systems.

### 4.2. Cell Cultures

Peritoneal mesothelial cells (PMCs) were isolated from omentum obtained from eight patients undergoing abdominal surgery (Bioethics committee consent no. 578/18) by digestion with 0.05% trypsin-0.02% EDTA (for 20 min at 37 °C). The cells were cultured in M199 medium supplemented with L-glutamine (2 mM), 10% fetal bovine serum (FBS), and antibiotics. Peritoneal fibroblasts (PFBs) were established from the same tissues via enzymatic digestion for an additional 40 min. These cells were grown in Ham’s F-12 medium with the same supplements as those used for mesothelial cells. PMCs were identified based on positive stainings of the D2-40 and HBME-1, whereas PFBs were identified according to the expression of FSP1. Cells used in experiments were derived from first to second passage to avoid any contamination with senescent cells.

The ovarian cancer cell line A2780 was purchased from the ECCC (Porton Down, UK) and grown in RPMI 1640 medium supplemented with L-glutamine (2 mmol/L), 10% FBS, and antibiotics. OVCAR-3 cells were obtained from ATCC (Rockville, MD, USA) and grown in RPMI 1640 medium supplemented with L-glutamine (2 mmol/L), 20% FBS, HEPES (10 mmol/L), sodium pyruvate (1 mmol/L), glucose (4500 mg/L), insulin (0.01 mg/mL), and antibiotics.

### 4.3. Malignant and Benign Ascites

Malignant ascites (MAs) were collected from eight patients with high-grade serous ovarian carcinoma (stage III and IV). The histopathology, grade, and stage of the tumors were assigned in accordance with the criteria set by the International Federation of Gynecology and Obstetrics. Control, benign ascites (BAs) were obtained from eight age-matched non-cancerous patients. The study was approved by an institutional ethics committee (Bioethics committee consent no. 578/18). Upon collection under sterile conditions, the fluids were centrifuged at 2500 rpm for 5 min, and the cell-free supernatants were stored in aliquots at −20 °C until required. During experiments, cells were exposed to 10% MAs/BAs for 72 h [8].

### 4.4. Cell Adhesion Assay

Cancer cells probed with 5 µM calcein-AM (Molecular Probes, Invitrogen, Eugene, OR, USA) for 30 min at 37 °C were plated (3 × 10^4^ cells/well) on top of monolayered PMCs or PFBs. After a 45-min incubation at 37 °C, the total fluorescence was quantified in a Synergy^TM^ 2 spectrofluorometer (BioTek Instruments, Winooski, VT, USA) using 485 nm and 535 nm excitation and emission wavelengths, respectively. Afterwards, nonadherent cells were removed by washing, and the fluorescence measurement was repeated. The second set of recorded values were compared to total fluorescence values to calculate the percentage of cells bound. In some experiments, adhesion was tested in the presence of MAs preincubated with neutralizing antibodies against EGF (0.03 µg/mL), GRO-1 (840 ng/mL), HGF (1000 ng/mL), IGF-1 (200 ng/mL), and TGFβ1 (400 ng/mL) for 4 h. In other assays, cells were treated for 72 h with exogenous, recombinant TGF-β1 (400 mg/mL), HGF (2500 pg/mL), GRO-1 (500 pg/mL), and IGF-1 (50 pg/mL) or preincubated with Cpd22 (1 µM) for 3 h prior to the application of MAs/BAs.

### 4.5. Immunofluorescence

The expression of vimentin and ICAM-1 was quantified using immunofluorescence. Cells were exposed to specific antibodies (1:500 dilution) overnight at 4 °C. Then, they were washed and treated with Alexa Fluor 488 (Invitrogen, Waltham, MA, USA) for 1.5 h at room temperature. The fluorescence was recorded using the Synergy^TM^ 2 device.

### 4.6. Flow Cytometry

The expression of α5β1 integrins was assessed by flow cytometry. Briefly, cells were harvested on ice and washed in ice-cold Hank’s balanced salt solution (HBSS). Then, the cells were incubated for 45 min on ice with either the anti-human integrin α5β1 monoclonal antibody or the isotype control antibody (1:500 dilution). After they were rinsed with ice-cold HBSS, cells were incubated for 45 min on ice with fluorescein-conjugated goat anti-mouse secondary antibody AlexaFluor 488 (Invitrogen; 1:1000 dilution). Cells were then washed and analyzed with a GUAVA EasyCyte 6HT-2L flow cytometer (Merck). The data obtained were analyzed and graphically presented using GuavaSoft v3.1.1 (Merck).

### 4.7. Statistics

Statistical analysis was performed using GraphPad Prism™ v.5.00 (GraphPad Software, San Diego, CA, USA). The means were compared with the Wilcoxon matched-pairs test or repeated ANOVA with a post hoc Newman–Keuls test when appropriate. The results are expressed as the means ± SEM. Differences with a *p*-value <0.05 were considered statistically significant.
